# Frog tongue surface microstructures: functional and evolutionary patterns

**DOI:** 10.3762/bjnano.7.81

**Published:** 2016-06-22

**Authors:** Thomas Kleinteich, Stanislav N Gorb

**Affiliations:** 1 Functional Morphology and Biomechanics, Zoology Department, Kiel University, 24118 Kiel, Germany

**Keywords:** adhesion, amphibians, biological materials, feeding, high-resolution micro-CT

## Abstract

Frogs (Lissamphibia: Anura) use adhesive tongues to capture fast moving, elusive prey. For this, the tongues are moved quickly and adhere instantaneously to various prey surfaces. Recently, the functional morphology of frog tongues was discussed in context of their adhesive performance. It was suggested that the interaction between the tongue surface and the mucus coating is important for generating strong pull-off forces. However, despite the general notions about its importance for a successful contact with the prey, little is known about the surface structure of frog tongues. Previous studies focused almost exclusively on species within the Ranidae and Bufonidae, neglecting the wide diversity of frogs. Here we examined the tongue surface in nine different frog species, comprising eight different taxa, i.e., the Alytidae, Bombinatoridae, Megophryidae, Hylidae, Ceratophryidae, Ranidae, Bufonidae, and Dendrobatidae. In all species examined herein, we found fungiform and filiform papillae on the tongue surface. Further, we observed a high degree of variation among tongues in different frogs. These differences can be seen in the size and shape of the papillae, in the fine-structures on the papillae, as well as in the three-dimensional organization of subsurface tissues. Notably, the fine-structures on the filiform papillae in frogs comprise hair-like protrusions (Megophryidae and Ranidae), microridges (Bufonidae and Dendrobatidae), or can be irregularly shaped or absent as observed in the remaining taxa examined herein. Some of this variation might be related to different degrees of adhesive performance and may point to differences in the spectra of prey items between frog taxa.

## Introduction

Frogs (Lissamphibia: Anura) are famous for their adhesive tongues, which allow them to catch elusive prey. While the movements of the tongue during feeding in different groups of frogs have received considerable attention in the past [1–6], little is known about the functional mechanisms for the adhesiveness of frog tongues. Obviously, adhesion is critical to secure the prey item and to move it into the mouth. In a previous study we demonstrated for South American horned frogs (genus *Ceratophrys*) that the adhesive forces that frog tongues can produce and withstand are even higher than the body weight of the animals, at least if measured against a glass surface [7]. Further, we found that adhesive forces are higher if less mucus remained on our test surface and that the amount of the mucus coverage increases with increasing contact duration. These results suggested (1) that during the initial contact formation, only small amounts of mucus are present on the tongue and (2) that besides chemical and physical properties of the mucus, other mechanisms at the interface between a frog tongue and a target will have an important impact on tongue adhesiveness [7].

Frog tongues are known to have two types of papillae on their surface. So-called fungiform papillae (type 1) are surrounded by numerous, smaller filiform papillae (type 2) [8]. The fungiform papillae are suggested to act as chemoreceptors, while the filiform papillae are the places for mucus production [9–12]. Owing to the fact that the filiform papillae cover wide parts of the adhesive tongue surface in frogs, the interaction between the filiform papillae, the mucus layer, and the target surface of a prey item will be critical for a successful feeding event. Thus, besides mucus production, the filiform papillae as surface microstructures might actually mediate adhesive performance. More recently we discussed the contribution of the filiform papillae to the adhesive mechanism of the tongue in the frog *Ceratophrys ornata* [13]. We suggested that the papillae increase the adaptability of the tongue to uneven surfaces and may help to form and anchor fibrils of mucus that emerge before a frog tongue is about to loose the contact with a target surface.

While it has been shown before, that the anatomy of frog tongues can be very diverse in different anuran taxa [14], little is known about the diversity of tongue surface structures in frogs. Besides a study on the ornamentation of the tongue in the dicroglossid frog *Fejervarya cancrivora* [15] (the frog is referred to as *Rana cancrivora* in that study), only the tongue surfaces in a few species of the genera *Rana* [15–21] and *Bufo* [22–24] have been described in the literature. Further accounts on tongues in *Hyla arborea* [9] and *Calyptocephalella gayi* [25] focus on the fungiform papillae but neglect the filiform papillae despite their presumably important role in tongue adhesion. Iwasaki (2002) [8] highlights notable differences in the filiform papillae between *Rana* spp. and *Bufo japonicus*. In *Rana* spp., the filiform papillae appear as hair-like structures, while in *B. japonicus*, the filiform papillae rather take the form of ridges. In a more recent study, however, Elsheikh et al. [24] described hair-like filiform papillae for another species within the Bufonidae, i.e., *Sclerophrys regularis* (in [24] as *Bufo regularis*).

Besides the surface profile and mechanical properties of the tongue surface, the tissues underlying the surface represent another important factor for the adhesive performance of the tongue. The configuration of subsurface tissues will determine how well a tongue can adapt to a target surface and how well the tongue withstands the forces that act during protraction and retraction. Besides our recent description of the inner anatomy of the tongue in *Ceratophrys ornata* [13], nothing is known about the three-dimensional architecture of the tissues underneath the tongue surface in frogs. This kind of data might shed light on the presence of potential gradients of the material stiffness that were previously described for attachment structures in beetles [26–27], grasshopers [28], and geckos [29].

Here we combine scanning electron microscopy and high-resolution micro-computed tomography (micro-CT) to provide comparative accounts on the surface profiles and subsurface structures of the tongues in nine different frog species. The aims of this study are: (1) to evaluate patterns of the diversity of tongue surfaces in frogs, (2) to provide descriptions on the three-dimensional organization of the tissues underneath the frog tongue surface, and (3) to understand patterns of tongue variation in frogs within both evolutionary and biomechanical contexts.

## Experimental

We studied the tongue anatomy of nine different species comprising eight of the currently 55 recognized taxa (families) within the Anura [30]. A list of specimens is provided in Table 1. The specimens were either made available by the Zoological Museum Hamburg (ZMH) or were derived from the uncatalogued stock of the Zoological Institute and Museum at Kiel University. In the latter case, we used our own specimen IDs (TK) herein. All specimens were stored in 70% ethanol. Two specimens actually belonged to the same genus but comprised two different species: *Litoria infrafrenata* and *L. caerulea*. As these two species appeared to be very similar in their tongue anatomy, they are referred to as *Litoria* spp. herein. The two specimens of *Ceratophrys ornata* have been examined in prior studies on feeding and tongue adhesion in frogs of the genus *Ceratophrys* [13,31]. The *Litoria caerulea* specimen studied herein was previously used for a study on toe-pad anatomy in tree frogs [32].

**Table 1:** Specimens examined herein.

taxon (family)	species	collection ID	SVL [mm]	method	voxel size µCT [µm]

Alytidae	*Discoglossus pictus*	ZMH A11869	48	SEM	—
Alytidae	*Discoglossus pictus*	ZMH A11885	48	µCT	0.67
Bombinatoridae	*Bombina variegata*	ZMH A11872	38	SEM	—
Bombinatoridae	*Bombina variegata*	ZMH A11873	37	µCT	0.67
Bufonidae	*Bufo bufo*	TK Bufo01	N/A	µCT & SEM	0.73
Ceratophryidae	*Ceratophrys ornata*	ZMH A11916	59	SEM	—
Ceratophryidae	*Ceratophrys ornata*	ZMH A11917	70	µCT & SEM	0.87
Dendrobatidae	*Oophaga histrionica*	ZMH A11874	29	µCT	0.87
Dendrobatidae	*Oophaga histrionica*	ZMH A11875	32	SEM	—
Hylidae	*Litoria caerulea*	TK Litoria01	68	µCT & SEM	0.53
Hylidae	*Litoria infrafrenata*	ZMH A11870	75	µCT & SEM	0.87
Megophryidae	*Megophrys nasuta*	ZMH A11865	71	SEM	—
Megophryidae	*Megophrys nasuta*	ZMH A11866	103	µCT	0.87
Megophryidae	*Megophrys nasuta*	ZMH A11868	68	µCT	0.67
Ranidae	*Rana* (*Lithobates*) *pipiens*	TK Rana01	N/A	µCT	1.13
Ranidae	*Rana* (*Lithobates*) *pipiens*	TK Rana02	N/A	SEM	—

We examined the surface structures of frog tongues by using scanning electron microscopy (SEM). For SEM we prepared pieces from the central regions of the tongues. These pieces were first dehydrated in an ascending series of ethanol (70%, 90%, 100%; each step was maintained for 24 h). For specimens that were also used for micro-computed tomography prior to SEM (see Table 1), two additional dehydration steps (30% and 50%) were necessary, as the micro-CT imaging was performed in distilled water. After dehydration, the tongue specimens were critical point dried with a Quorum E3000 critical point drying system (Lewes, UK). Then the tongue specimens were mounted with the dorsal side facing upwards onto aluminum stubs using carbon-containing double-sided adhesive tape. The specimens were then coated with a 10 nm gold–palladium layer by using a Leica SCD05 Sputter Coater (Leica Microsystems GmbH, Wetzlar, Germany). For scanning electron microscopy, we used a Hitachi S-4800 scanning electron microscope at an accelerating voltage of 3 kV (Hitachi High-Technologies Europe GmbH, Krefeld, Germany).

We used micro-computed tomography (micro-CT or µCT) to study the three-dimensional arrangement of tissues underneath the tongue surface. To visualize soft tissue structures, such as the epithelium and muscle fibers, we stained the frogs with 4% Lugol’s iodine potassium iodide solution before we dissected the tongues. For this purpose, we followed the protocol by Metscher [33] but adjusted the staining duration to two weeks to allow the staining solution to diffuse deep into entire frog specimens. After staining, we dissected the tongues and cut out pieces that were approximately 1.5 mm × 1.5 mm × 2 mm (length × width × height) from the dorsal surface in central regions of the tongue. These pieces were then placed into the tips of pipettes that we filled with distilled water. To prevent leakage during the scan, we wrapped the pipette tips with laboratory film (Parafilm M**^®^**, Bemis Company Inc., Oshkosh, WI, USA). We then mounted the pipette tips with the tongue specimens into a Skyscan 1172 desktop micro-CT scanner (Bruker microCT, Kontich, Belgium). We operated the micro-CT scanner with a source voltage of 40 kV and a current of 250 µA. The small size of the specimens allowed us to fit the pieces of tongue tissue into a very narrow field of view (<2 mm) during the scan, which corresponds to the maximal magnification of the Skyscan 1172 and resulted in voxel sizes of less than 1 µm (Table 1). From the X-ray images, captured during micro-CT scanning, we reconstructed image stacks of virtual cross-sections through the entire specimen with the software NRecon (Bruker microCT, Kontich, Belgium). These image stacks were then exported as 16 bit TIFF files, which we analyzed and visualized with the 3D visualization software package Amira 6.0 (FEI SAS, Mérignac Cedex, France). The micro-CT data of the *Ceratophrys ornata* specimen was already used in a previous study [13] and is accessible at http://dx.doi.org/10.5061/dryad.066mr.

## Results

**Figure 1:**
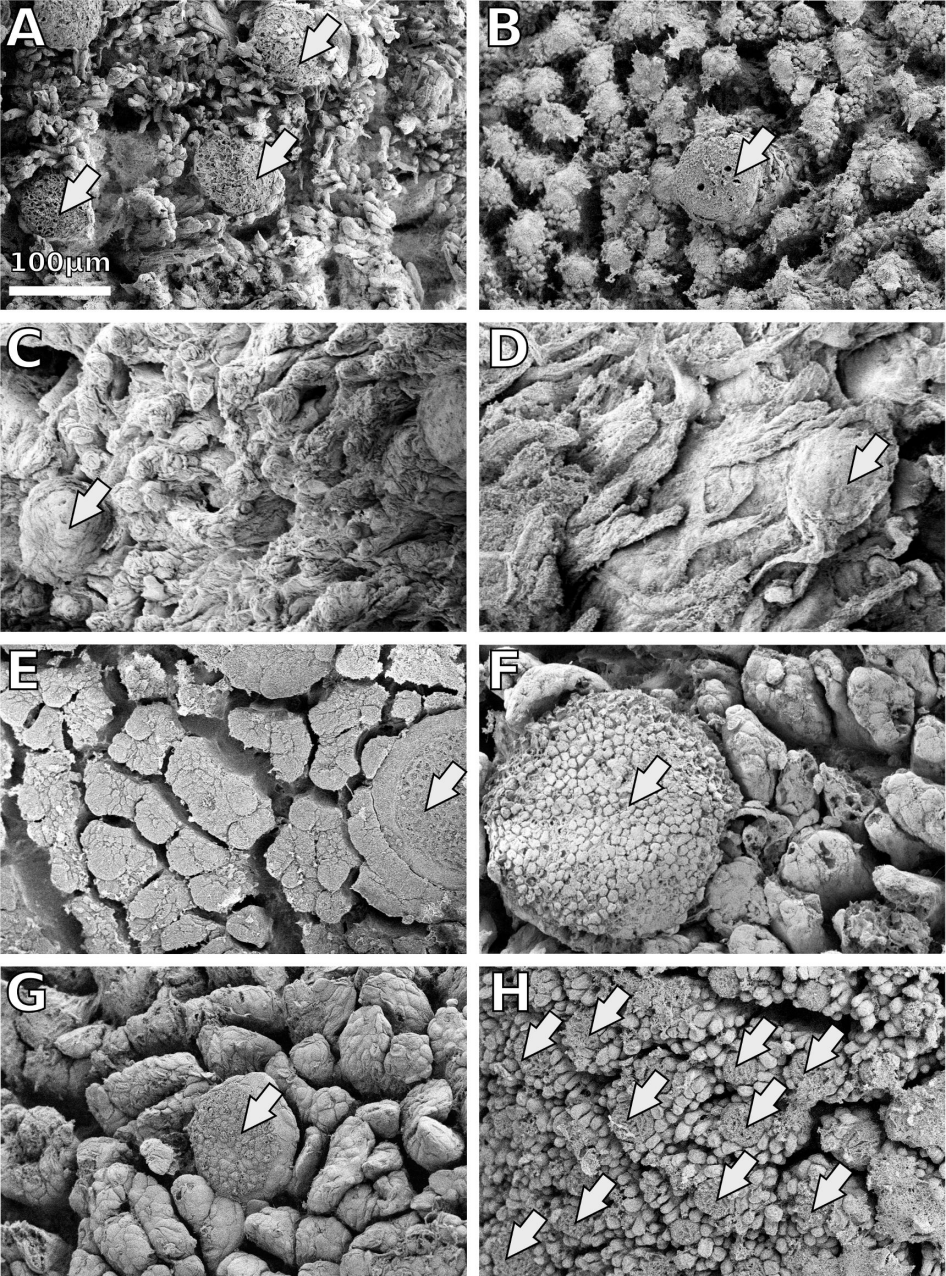
Scanning electron microscopy of frog tongue surfaces. All images are at the same scale. A – *Bombina variegata*, B – *Discoglossus pictus*, C – *Ceratophrys ornata,* D – *Litoria infrafrenata*, E – *Megophrys nasuta,* F – *Rana (Lithobates) pipiens*, G – *Bufo bufo*, H – *Oophaga histrionica*. Frog tongue surfaces are covered by fungiform papillae (arrows), which are embedded in a matrix of smaller filiform papillae. The examined species differ notably in the size and the shape of the papillary surface structures.

**Tongue surface structures:** Two types of papillae cover the dorsal surface of frog tongues: numerous filiform papillae build a matrix in that the larger fungiform papillae are embedded (Figure 1). We observed notable interspecific differences in the size and shape of these papillae. The fungiform papillae have roughly the same diameter of 70 to 90 µm in the *Bombina variegata*, *Discoglossus pictus, Ceratophrys ornata, Litoria* spp. (Figure 1A–D), and *Bufo bufo* (Figure 1G) specimens examined (Table 2). However, in *Megophrys nasuta* (Figure 1E) and *Rana (Lithobates) pipiens* (Figure 1F), the fungiform papillae appear larger than in the remainder species. In *Oophaga histrionica,* the fungiform papillae are smaller (Figure 1H; Table 2).

**Table 2:** Measurements of tongue papillae in micrometers based on Figure 1.

	*Bombina variegata*	*Discoglossus pictus*	*Ceratophrys ornata*	*Litoria infrafrenata*	*Megophrys nasuta*	*Rana (Lithobates) pipiens*	*Bufo bufo*	*Oophaga histrionica*

fungiform papillae	62.87	79.72	78.24	85.27	125.00	216.15	87.69	30.29
	69.98	—	78.19	81.73	—	—	—	29.34
	67.73	—	—	—	—	—	—	27.55
average	66.86	79.72	78.22	83.50	125.00	216.15	87.69	29.06

filiform papillae	8.46	5.47	21.39	16.47	19.86	52.19	32.56	8.99
	6.02	6.02	19.19	17.46	21.08	51.77	34.04	9.64
	7.00	8.77	23.93	19.28	25.41	51.47	34.88	8.19
	7.09	7.14	19.88	13.58	18.27	43.50	35.03	8.25
	6.60	6.34	14.89	12.71	20.91	53.93	32.00	8.01
	5.94	7.21	22.33	11.38	20.86	38.35	31.07	7.96
average	6.85	6.83	20.27	15.15	21.07	48.54	33.26	8.51

The filiform papillae appear as rod-like protrusions with a diameter of approximately 7 µm in *Bombina variegata*, *Discoglossus pictus*, and *Oophaga histrionica*. In *Ceratophrys ornata*, the filiform papillae also appear rod-like, but with a thicker diameter of 20 μm and a lower aspect ratio (Figure 1C). In *Megophrys nasuta*, *Rana (Lithobates) pipiens*, and *Bufo bufo*, the filiform papillae are thicker than in the remainder species, and clutches of filiform papillae form ridge-like structures (Figure 1E–G). In *Litoria* spp., unlike any other species studied herein, the filiform papillae were found to be elongated and hair-like (Figure 1D). The terminal parts of the filiform papillae in frog tongues have a rounded shape except for *M. nasuta*, in which the filiform papillae have flat tips (Figure 1E).

**Figure 2:**
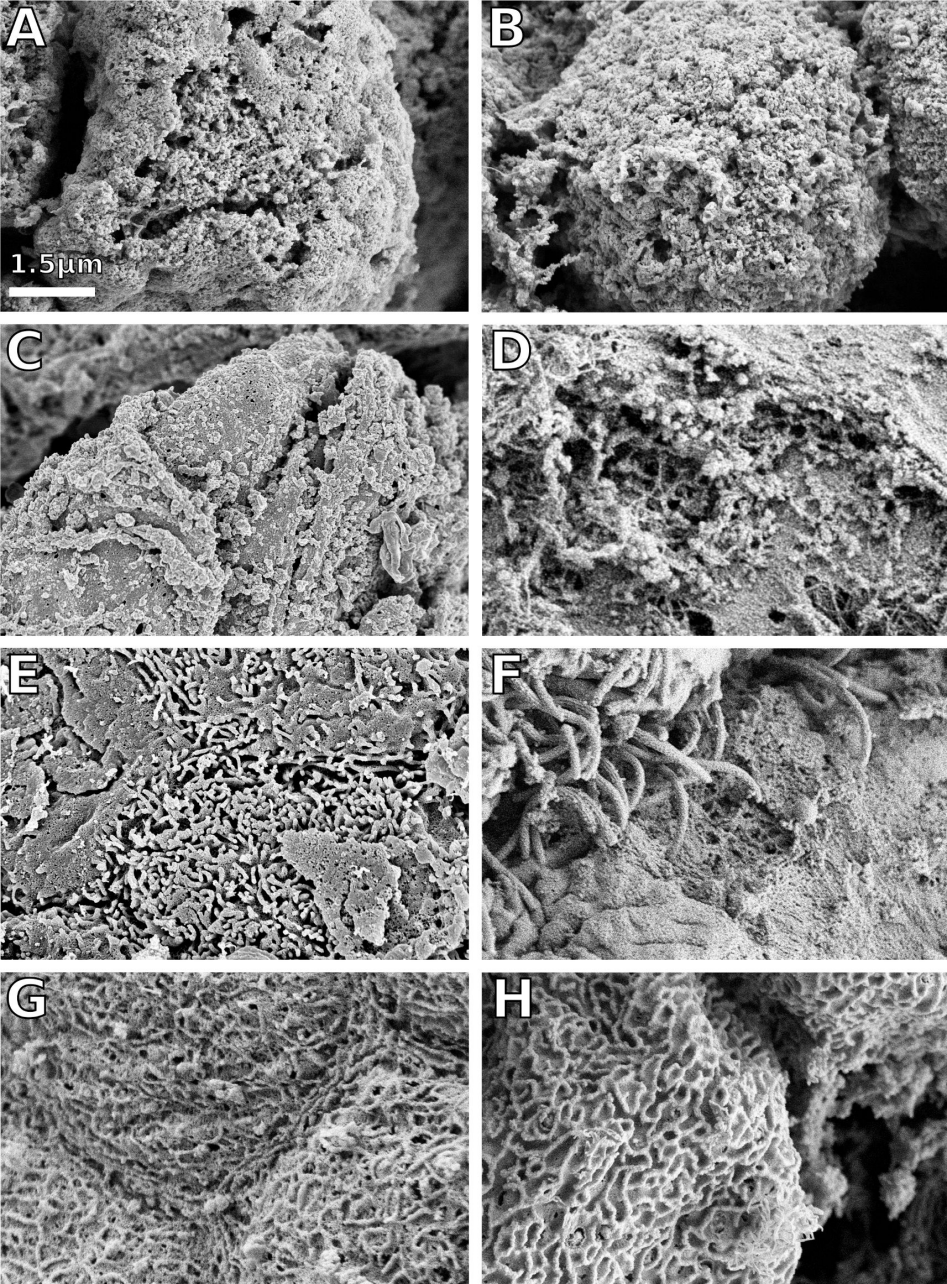
Scanning electron microscopy of the filiform papillae on frog tongues. All images are at the same scale. A – *Bombina variegata*, B – *Discoglossus pictus*, C – *Ceratophrys ornata*, D – *Litoria infrafrenata*, E – *Megophrys nasuta*, F – *Rana* (*Lithobates) pipiens*, G – *Bufo bufo*, H – *Oophaga histrionica*. The filiform papillae show a remarkable degree of interspecific variation. In *M. nasuta* (E) and *R. pipiens* (F), we found hair-like outgrowths on the filiform papillae; in *B. bufo* (G) and *O. histrionica* (F), the filiform papillae are covered by micro-ridges.

Further we found interspecific variation in the surface patterns of the filiform papillae (Figure 2). In *Megophrys nasuta*, the tips of the filiform papillae are covered by hair-like protrusions, which were approximately 80 nm in diameter (Figure 2E). In *Rana* (*Lithobates*) *pipiens*, hair-like protrusions are also present on the filiform papillae. However, these structures in *R. pipiens* are much larger compared to *M. nasuta* and appear only in patches (Figure 2F). The filiform papillae in *Bufo bufo* and *Oophaga histrionica* differ from the remainding species examined by being covered with nanoscale surface ridges (Figure 2G,H). In *Bombina variegata*, *Discoglossus pictus*, *Ceratophrys ornata*, and *Litoria* spp., no notable surface structures could be observed. The surfaces of the filiform papillae in these species appear irregularly shaped (Figure 2A–D).

**Figure 3:**
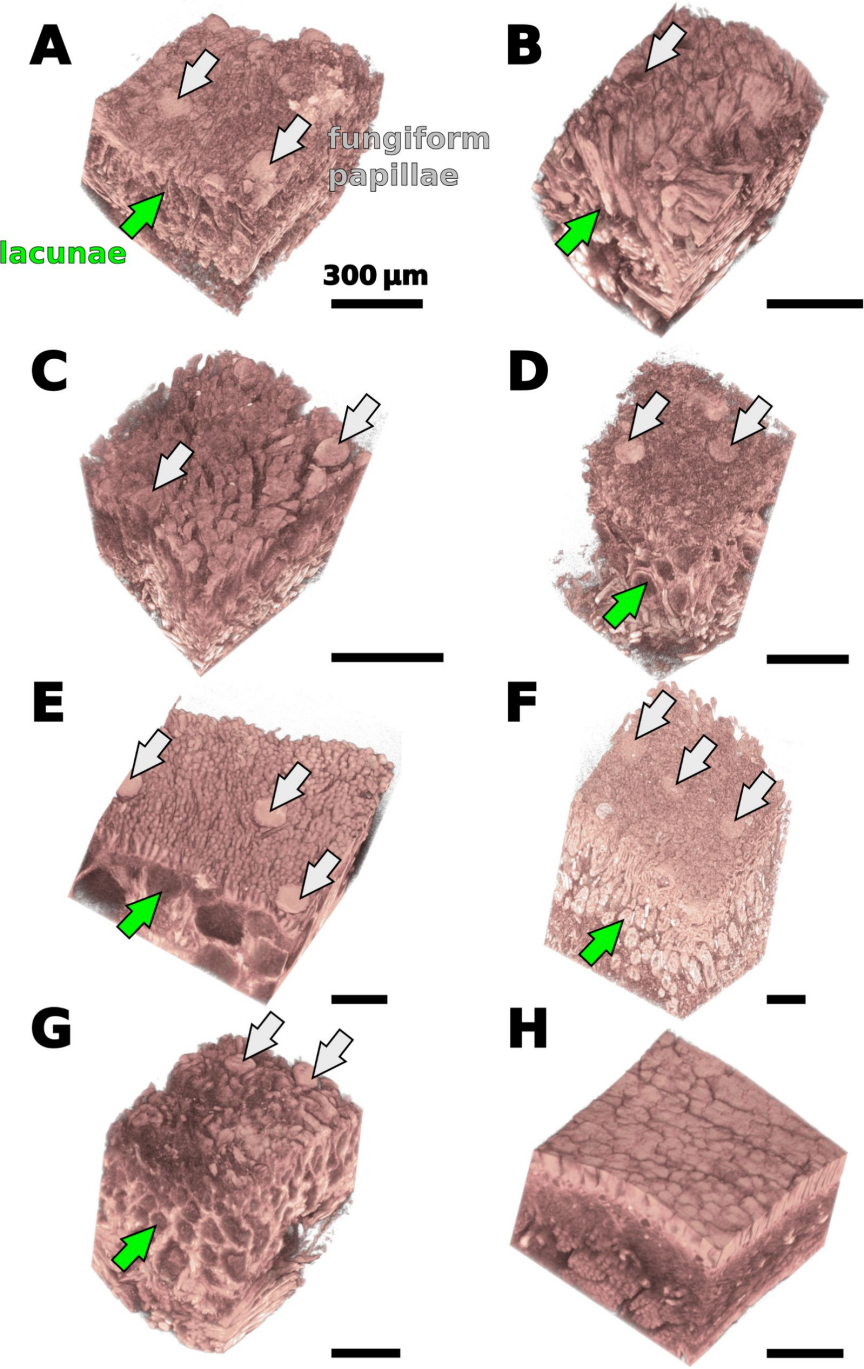
Micro-CT images of tissue fragments that were derived from the surfaces of frog tongues. A – *Bombina variegata*, B – *Discoglossus pictus*, C – *Ceratophrys ornata*, D – *Litoria caerulea*, E – *Megophrys nasuta*, F – *Rana* (*Lithobates*) *pipiens*, G – *Bufo bufo*, H – *Oophaga histrionica*. Except for *C. ornata* (C) and *O. histrionica* (H), the fungiform papillae (grey arrows) can easily be identified in the micro-CT data. Underneath the papillary surface structures lies a layer with lacunar structures that appear hollow in the micro-CT scan (green arrows). Both size and shape of these lacunae strongly vary among different species.

**Figure 4:**
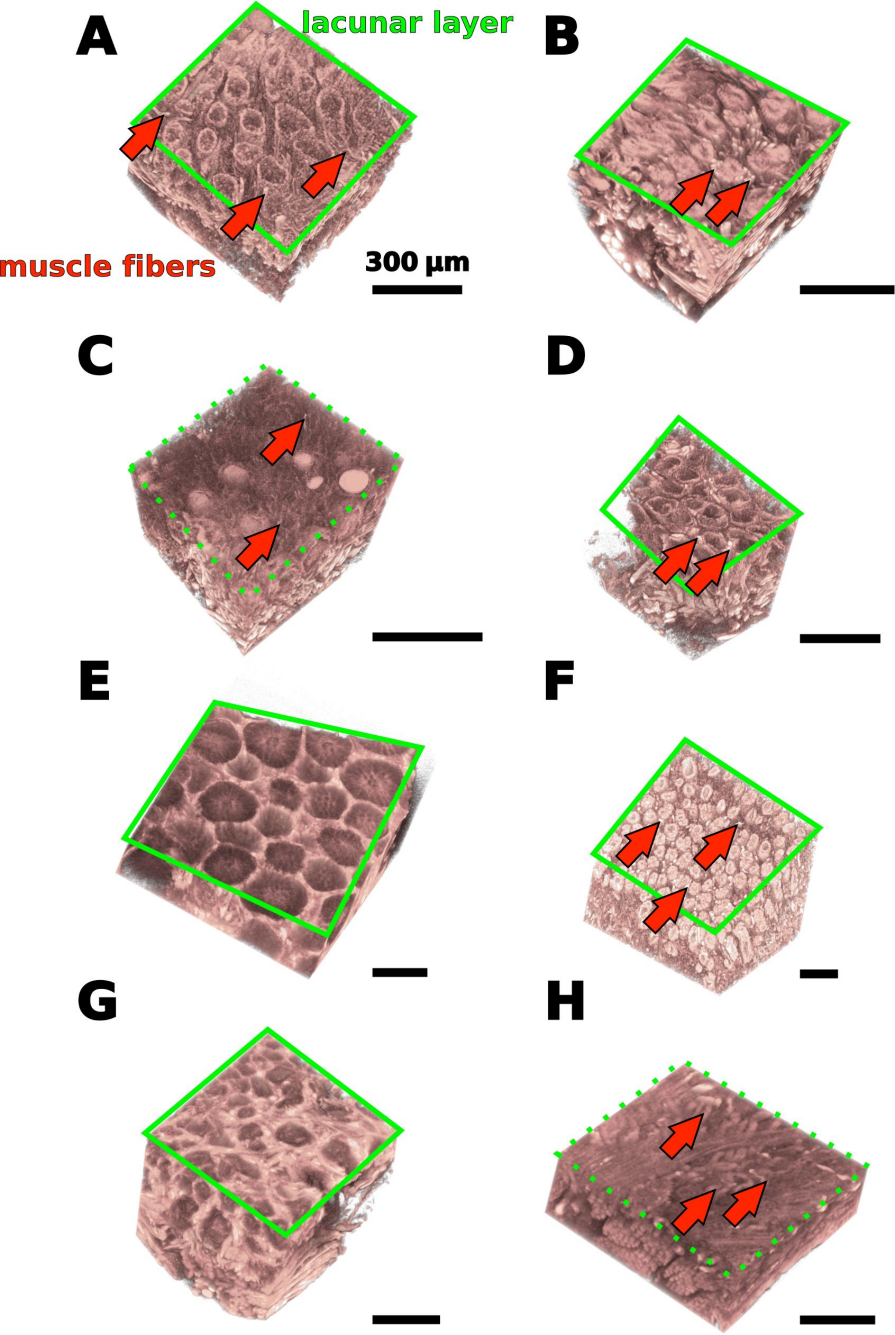
Virtual section through the micro-CT data of tongue tissue fragments at the level of the lacunar layer. A – *Bombina variegata*, B – *Discoglossus pictus*, C – *Ceratophrys ornata*, D – *Litoria caerulea*, E – *Megophrys nasuta*, F – *Rana* (*Lithobates*) *pipiens*, G – *Bufo bufo*, H – *Oophaga histrionica*. In *B. variegata* (A), *D. pictus* (B), and *L. infrafrenata* (D), the lacunae seem to be more similar than in the remainder species. In *C. ornata* (C) and *O. histrionica* (H), we were not able to find lacunae by using micro-CT imaging. Full slice movies of the micro-CT data are available in Supporting Information File 1. Muscle fibers (red arrows) emerge between the lacunae and face towards the tongue surface.

**Three-dimensional organization of the tongue tissue:** Contrast enhanced high-resolution micro-CT imaging allowed us to visualize the three-dimensional organization of subsurface soft tissue structures in frog tongues (Figure 3 and Figure 4). The resulting voxel sizes for the micro-CT datasets ranged from 0.53 to 1.13 µm (Table 1). With this spatial resolution, we were able to identify structures in the micrometer scale, such as the filiform and fungiform papillae (Figure 3). In *Oophaga histrionica*, however, the spatial resolution of the micro-CT dataset was not sufficient to discriminate between the two types of surface papillae (Figure 3H).

Underneath the surface papillae, we found a layer of lacunar structures that in the micro-CT data appear to be almost hollow inside (Figure 4; Supplementary movies). In *Bombina variegata, Discoglossus pictus, Litoria* spp., and *Rana (Lithobates) pipiens*, these lacunae are elongated and of cylindrical shape. Muscle fibers of the tongue musculature emerge between these cylinders towards the tongue surface (Figure 4; movies in Supporting Information File 1). In *Megophrys nasuta* and *Bufo bufo*, the lacunar structures appear to be more spherical than in *B. variegata, D. pictus, Litoria* spp., and *R. pipiens*. Further, in *M. nasuta*, the lacunae are stacked in two, in *B. bufo* even in up to four layers (movies 5 and 7 in Supporting Information File 1). In the *Ceratophrys ornata* specimen, the layer underneath the surface papillae was only poorly stained in the micro-CT scan and lacunae are not visible. However, in a deeper layer, spherical structures can be seen that differ from the hollow lacunae in the remainder species and show a strong X-ray absorption contrast (Figure 4C). Muscle fibers run in-between these spherical structures towards the dorsal surface of the tongue in *C. ornata*. In *Oophaga histrionica*, we were not able to identify lacunae underneath the surface papillae by using micro-CT imaging. Underneath the layer consisting of lacunar, respectively spherical structures in the case of *Ceratophrys ornata*, bundles of tongue muscle fibers are arranged parallel and perpendicular to the tongue surface (movie 3 in Supporting Information File 1). In *Oophaga histrionica*, the fibers of the tongue musculature appear to run directly underneath the surface papillae of the tongue (Figure 4H, movie 8 in Supporting Information File 1).

## Discussion

Here we demonstrate a high degree of interspecific variation in the surface anatomy of frog tongues. Differences can be seen in the arrangement of tissue layers close to the tongue surface and in the density, shape, and surface profiles of the filiform papillae. Although we lack experimental data on tongue performance for most of the species discussed herein, we hypothesize that these differences are likely to have effects on the adhesive and frictional properties of the tongues in frogs.

Especially the numerous filiform papillae may play a key-role in tongue adhesion besides their function in mucus production. The papillae themselves deform under compression and thus help to make the tongue adaptable to surface asperities of the prey item. In *Megophrys nasuta* and *Rana (Lithobates) pipiens*, where we found hair-like structures on top of the filiform papillae. This second level of hierarchical organization is hypothesized to increase the adaptability of the tongue under load. The micro-ridges on the filiform papillae in *Bufo bufo* and *Oophaga histrionica* and the irregular surface structures in *Bombina variegata, Discoglossus pictus, Ceratophrys ornata*, and *Litoria* spp. probably will not deform as much under compression as the hair-like structures in *M. nasuta* and *R. pipiens*. Thus, in *B. variegata, D. pictus, C. ornata*, and *Litoria* spp., the adaptability of the tongue to surface asperities of the prey will only depend on the filiform papillae themselves.

It seems reasonable to assume that species that form and maintain a better contact with the prey surface are more likely to generate high adhesive forces during tongue feeding. Other than for *Ceratophrys* sp. [7], we have no force data on tongue adhesion available at this time. However, another measure for tongue adhesive performance might be the sizes of typical prey items as these are captured with the tongues. *Bufo bufo* and *Oophaga histrionica* are known to feed on relatively small prey, such as ants [34–37], while *M. nasuta* and *R. pipiens* are considered to be generalist feeders capturing a wide variety of prey items of different sizes and even preying upon small vertebrates [36]. This picture, however, becomes much more complicated if one also considers the other species examined herein. Especially frogs within the genus *Ceratophrys* are known to be voracious generalist feeders [31,38–40] and their tongues can produce notable adhesive forces [7]. However, the *C. ornata* examined in the work described herein lacks hair-like outgrowths on its filiform papillae and, therefore, its tongue might be less adaptable to the prey surface than those of *M. nasuta* or *R. pipiens*.

We previously argued that the filiform papillae also interact with the mucus covering the tongues in live frogs [13] (see also Sperry and Wassersug [41]). Frog tongues might thus be considered as composite structures of mucus plus papillae. The size, aspect ratio, and distribution of the papillae will have an impact on how this composite is stabilized. Denser arrays of surface papillae are likely to improve the cohesion within the mucus-papillae composite, and thus prevent failure of the mucus layer during tongue retraction. It is plausible to assume that different species have different physical (rheological) properties of the mucus, and that these properties are correlated with a particular microstructure of the tongue or vice versa. The viscosity of the mucus is critical for attachment especially given the short time frames between tongue impact and retraction, which happens within milliseconds [5–7,42]. The rapidness with which mucus can wet a target surface will decrease with increasing viscosity. Denser arrays of filiform papillae might allow for less viscose mucus that is still stable enough to withstand the tongue pulling forces. Based on the size of the filiform papillae (Figure 1), we would therefore expect for our specimen sample that *Oophaga histrionica* has the least viscous mucus, while *Bufo bufo*, *Rana* (*Lithobates*) *pipiens*, and *Megophrys nasuta* are predicted to have the most viscous mucus. This hypothesis, however, remains to be tested in future studies.

Besides the profile of the surface, also the composition of the underlying tissues will have an impact on the adhesive performance of the tongue. Such a role of deeper tissue layers, which influence the compliance of the adhesive structure, has recently been shown for the feet of grasshoppers [28] and geckos [29]. Although we did not measure the material attributes herein, the architecture of the subsurface layers might suggest the presence of a material gradient in frog tongues as well. It seems reasonable to assume that the mucus coverage on the tongue surface is more compliant than the filiform papillae themselves, which are in turn more flexible than the lacunar sub-surface layer. The muscle fibers underneath the lacunar layer supposedly have even higher stiffness than the lacunar layer. Such material gradients can be beneficial for adhesion as they will allow for a high adaptability to a target surface profile, while maintaining mechanical stability [26–27] and integrity of layered tissues.

The lacunar sub-surface layer that we describe herein for frog tongues is very similar in the three species that use mechanical pulling for feeding (as defined by Nishikawa [6]), i.e., *Bombina variegata, Discoglossus pictus,* and *Litoria* spp. but very diverse in the remainder species that use tongue projection. During mechanical pulling, the tongue deforms by action of the tongue musculature and is slightly protracted over the tip of the lower jaw [6,42]. However, other than during tongue projection, the tongue is not passively elongated by inertia. A more complete taxon sampling and evaluation of the physical properties of the lacunar layer in different frog tongues are needed to test if there is a mechanical benefit of cylindrical lacunae for mechanical pulling.

Further, it is important to keep in mind the limitations of micro-CT imaging here. The voxel sizes of the tongue surface scans were less than one micrometer. However, to visualize structures, several connected voxels are needed. Thus the spatial resolution of our approach is more likely in the range of three to four micrometers. Structures that are in the sub-micrometer range, such as the hair-like extrusions on top of the filiform papillae in *Megophrys nasuta*, therefore cannot be detected with the micro-CT. The absence of lacunae in *Ceratophrys ornata* and *Oophaga histrionica*, might be an artifact that is caused by a limited spatial resolution of our micro-CT setup. Further, although the lacunae generally appear hollow in the micro-CT scans, they might be filled with structures that could either be too thin to be detected with the micro-CT, that are X-ray transparent, or that cannot be stained by LUGOL’s solution. For *Pelophylax porosus*, a close relative to *Rana* (*Lithobates*) *pipiens*, Iwasaki et al. [12] prepared histological sections of the tongue and according to Figure 1 in their publication, the small lacunar structures in *L. pipiens* we describe herein relate to alveolar salivary glands. For *C. ornata,* we prepared fractured pieces of a critical point dried tongue and examined them with the SEM and also did not find lacunar spaces [13]. Future studies on the comparative histology of frog tongues will shed more light into this variation of the tongue at the microscopic level. The benefit of micro-CT imaging, however, is the immediate availability of the three-dimensional architecture of the investigated structures, which cannot be provided with other methods.

Despite the adhesive and frictional properties, also evolutionary relationships between the species examined might provide explanations for the interspecific variation we found herein. The tongues in *Discoglossus pictus* and *Bombina variegata* are rather similar, if compared to the remainder species examined herein. The two taxa, to which *D. pictus* and *B. variegata* belong to, i.e., the Alytidae and Bombinatoridae are widely considered as sister groups within anuran phylogeny [43–45]. Furthermore, *Bufo bufo* (Bufonidae) and *Oophaga histrionica* (Dendrobatidae) are closely related as the Bufonidae were either found to be the sister taxon to the Dendrobatidae [44–45] or to the Dendrobatidae plus species within the genus *Thoropa* [43]. The micro-ridges on the surface of the filiform papillae in *B. bufo* and *O. histrionica* are thus likely to be homologous and might represent a synapomorphy of the Bufonidae and Dendrobatidae. Similar ridges were previously described for a second species within the Bufonidae, i.e., *Bufo japonicus* [22].

However, besides the presence of micro-ridges on the surface of the filiform papillae, the tongues in *Bufo bufo* and *Oophaga histrionica* differ notably in their arrangement of the surface papillae and their three-dimensional organization. While the presence of micro-ridges in these two species might be explained by a close phylogenetic relationship of the Bufonidae and Dendrobatidae, the variation found in the shape and arrangement of the surface papillae and tongue tissue layers in these two species seems to be manifested at a smaller taxonomic scale. Further, the tongues of *Megophrys nasuta, Rana (Lithobates) pipiens, Litoria* spp.*,* and *Ceratophrys ornata* all have unique characters, such as the hair-like protrusions on the flat tips of the filiform papillae in *M. nasuta*, the patches of hairs on the filiform papillae in *R. pipiens*, the shape of the filiform papillae in *Litoria* spp. and the presence of X-ray dense spherical subsurface structures in *C. ornata*. Our taxon sampling herein proves insufficient to trace these unique tongue characteristics through anuran phylogeny. Many questions regarding the evolutionary and functional implications of this structural diversity remain and certainly inspire future research endeavors.

## Supporting Information

Supporting Information features a ZIP file containing eight slice movies through the micro-CT datasets, one for each of the following species: *Bombina orientalis* (Supplementary_Movie_01_Bombina.mov), *Discoglossus pictus* (Supplementary_Movie_02_Discoglossus.mov), *Ceratophrys ornata* (Supplementary_Movie_03_Ceratophrys.mov), *Litoria caerulea* (Supplementary_Movie_04_Litoria.mov), *Megophrys nasuta* (Supplementary_Movie_05_Megophrys.mov), *Rana* (*Lithobates*) *pipiens* (Supplementary_Movie_06_Rana.mov), *Bufo bufo* (Supplementary_Movie_07_Bufo.mov) and *Oophaga histrionica* (Supplementary_Movie_08_Oophaga.mov).

File 1 Slice movies through the micro-CT datasets of eight different frog species.
